# Distinguishing anthropogenic and natural contributions to coproduction of national crop yields globally

**DOI:** 10.1038/s41598-021-90340-1

**Published:** 2021-05-24

**Authors:** Matthias Schröter, Lukas Egli, Lilith Brüning, Ralf Seppelt

**Affiliations:** 1 Department of Computational Landscape Ecology, Helmholtz Centre for Environmental Research - UFZ, Permoserstr. 15, 04318 Leipzig, Germany; 2 Institute of Geoscience and Geography, Martin-Luther University Halle-Wittenberg, 06099 Halle (Saale), Germany

**Keywords:** Environmental sciences, Agroecology, Ecosystem services

## Abstract

Crop production is a crucial ecosystem service that requires a combination of natural and anthropogenic contributions to high and stable yields, which is a coproduction process. We analysed this coproduction based on nationally aggregated data for 15 major crops for 67 countries and the European Union with data for four time steps (2000, 2006, 2010, 2014). We found strong increases in fertilizer use, net capital stock and manure use intensity for lower-middle-income countries and stagnation or decrease of these for high-income countries. We used a multiple linear regression model predicting yield to distinguish the effect of anthropogenic contributions (crop-specific fertilizer use intensity, net capital stock intensity, manure use intensity) and natural contributions (crop-specific agricultural suitability, including soil characteristics, topography and climate). We found that in particular fertilizer use intensity, manure use intensity and agricultural suitability explained variation in yields to a considerable degree (R^2^ = 0.62).

## Introduction

Yields of food, feed and energy crops have rapidly increased over the past decades in many areas^1,2^. Crop production is a crucial ecosystem service that is provided as a result of a combination of natural and anthropogenic contributions that establish a coproduction process^3,4^. Crop yield is, despite being regularly used as an ecosystem service indicator^5,6^ not a well-suited measure for natural contributions to human well-being, as crop yield is only partly related to what agricultural ecosystems contribute and as it strongly depends on human management^7,8^. For instance, in a case study on accounting for natural contributions for six arable crops, Remme et al.^9^ found that the resource rent, as a proxy for natural contributions, only accounts for 12% of the total revenue of six arable crops, while operating, labour and capital costs account for the rest. On the one hand, crop production depends on a range of natural contributions such as soil formation and nutrient cycling leading to soil fertility^10,11^. Other important ecosystem services that contribute to crop production include animal pollination and biological pest control^12,13^. Abiotic natural aspects that influence agricultural suitability include topography and climate^14^. On the other hand, the importance of anthropogenic contributions for producing crops has been highlighted frequently^1^. One key aspect of anthropogenic contributions is an increasing intensity of land use through use of fertilizers, pesticides, fuel and machinery^1,15^. The framework of the Intergovernmental Science-Policy Platform on Biodiversity and Ecosystem Services (IPBES^16^) prominently features coproduction as the interplay of anthropogenic and natural contributions in creating ecosystem services. Little is known, however, about how to distinguish anthropogenic and natural contributions to yield of food, feed and energy crops, how relatively important these are and how they interact^3,13,17^.

Land use intensity trajectories, i.e. the development pathways of land use intensity over time, might characterize the change in the way agricultural products are coproduced. Such trajectories have been shown to differ across countries and regions with different development stages^1,18^ and generally indicate agricultural transitions from smallholder, subsistence farming to intensive agriculture^19^. According to trajectory theories, yields are generally expected to increase over time (while increases slow down^20^), and conventional intensification is expected to lead to a lower energy return on investment, i.e. the amount of energy gained from yield (caloric value of crops) as a proportion of energy invested in anthropogenic input (machinery, fuels, fertilizer)^21^. These overall changes in agricultural systems question to what extent natural contributions can be replaced by anthropogenic contributions when producing crops^22^. Fitter^22^, for instance, pointed out that anthropogenic capital could substitute (replacing the ecosystem service nutrient cycling through chemical fertilizers) or enhance (making nutrient cycling and primary productivity more available through technology) ecosystem services in agricultural production. Substitutability of natural contributions (or capital) is a key question in sustainability research^23,24^. Substitutability of natural through man-made capital would be in line with the weak sustainability position (which could imply some types of capitals could decrease) while no substitutability would be in line with strong sustainability assumption (no substitution implies the need to conserve all types of capital).

The aims of this study are twofold. First, we identified trajectories of anthropogenic contributions to coproduction of agricultural crops. For this purpose we quantified yield and three anthropogenic contributions of 15 crops over the period 2000–2014 on a national scale: crop-specific fertilizer use, manure applied to soils, and net capital stock in agriculture for three country income groups. Second, we modelled national yield globally with a set of explanatory variables to disentangle the effect of different anthropogenic contributions (fertilizer use intensity, net capital stock intensity, manure use intensity) and natural contributions (agricultural suitability, including soil characteristics, topography and climate^14^) over the same time period. We hypothesised both natural contributions and anthropogenic contributions to have positive effects on yields, but also expected interaction effects indicating substitution between natural and anthropogenic contributions, i.e. the higher the application of fertilizer or manure the lower the effect of agricultural suitability on yield or, the higher agricultural suitability the lower the effect of the application of fertilizer/manure. We hypothesised that the interaction between net capital stock in agriculture and agricultural suitability could show either an enhancement effect, i.e. the higher the amount of investment in agriculture, the higher the effect of natural contributions to yield due to a more effective use of natural resources, or a substitution effect as net capital stock might positively correlate with the other anthropogenic contributions and be indirect drivers of yield.

## Results

### Trajectories of anthropogenic contributions

**Figure 1:**
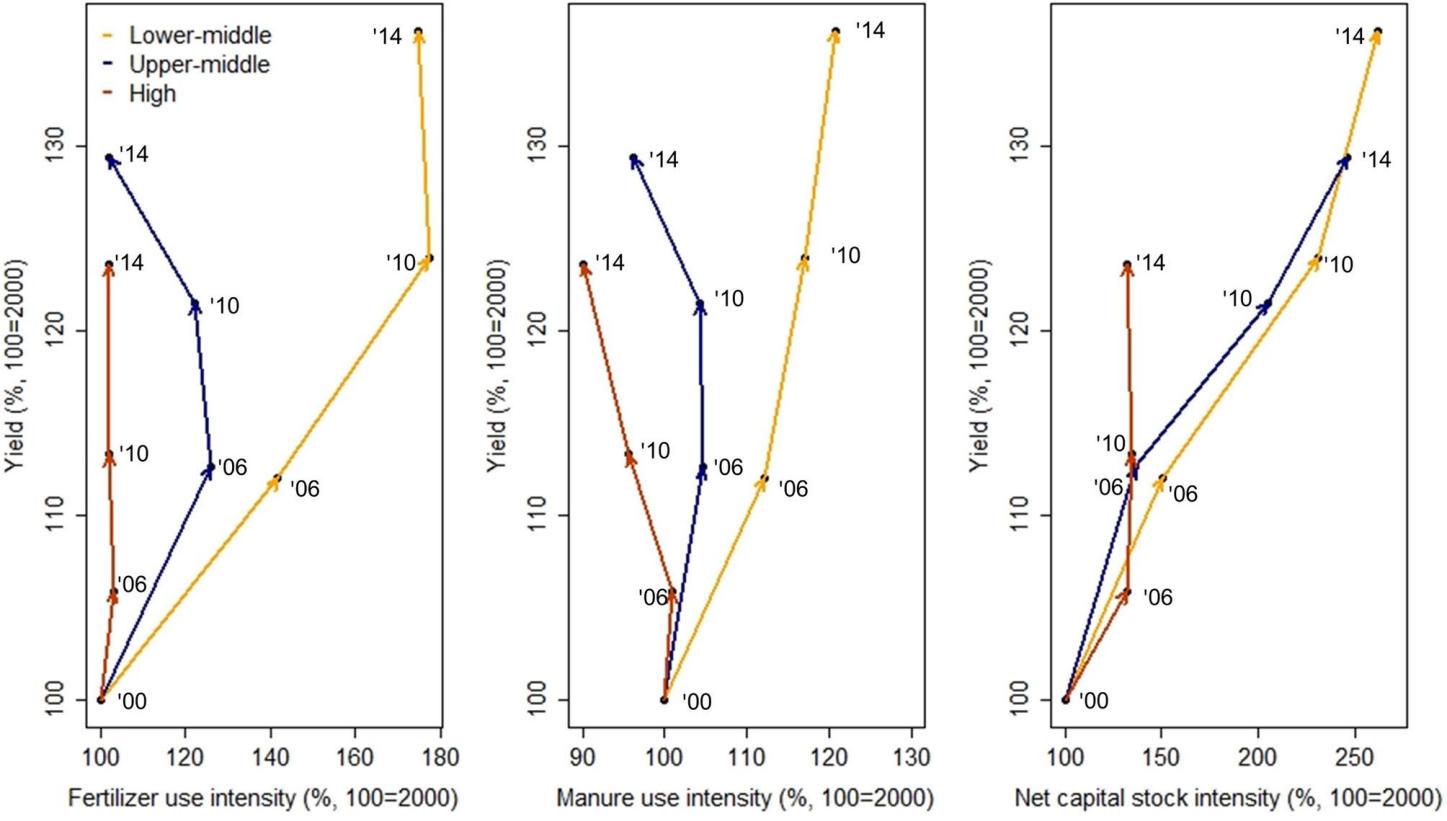
Trajectories of three anthropogenic coproduction factors for crop production in three Worldbank income classes (“lower-middle”, “upper-middle”, “high” income): fertilizer use intensity, manure use intensity and net capital stock intensity. Values are relative to the values in the year 2000 (100%). First arrow: 2000 to 2006, second arrow: 2006 to 2010, third arrow: 2010 to 2014. Yield increases over the years across income groups come along with stagnating or decreasing anthropogenic contributions in high income countries, and strong increase in net capital stock intensity in lower-middle and upper-middle income countries. Figure was created in R (version 3.6.1, https://www.r-project.org).

We identified trajectories for three anthropogenic contributions vs. yield of the 15 crops aggregated over the four time steps between 2000 and 2014 for three different income groups (Fig. 1). Yield increased most in lower-middle-income countries (aggregated over all countries: 36.2% in 2014 compared to 2000, mean across countries: 58.0%, range across countries: − 0.03–252.7%), followed by upper-middle (aggregated: 29.4%, mean: 30.4%, range: 20.8–44.4%) and high-income countries (aggregated: 23.6%, mean: 25.5%, range: 7.3–44.5%).

Fertilizer use intensity increased most in lower-middle-income countries over the considered time period (aggregated: 74.9%, mean: 57.3%, range: 6.6–159.8%), followed by upper-middle-income countries (aggregated: 2.2%, mean: 31.9% range: − 0.9–53.2%) and high-income countries (aggregated: 2.1%, mean: 30.7%, range: − 4.5–106.2%).

Manure use intensity showed an increase over the time period 2000–2014 only for lower-middle-income countries (aggregated: 20.7%, mean: 24.0%, range: − 2.4–55.7%), while it decreased in upper-middle-income countries (aggregated: − 3.8%, mean: 12.1%, range: − 22.8–37.4%) and in high-income countries (aggregated: − 9.9%, mean: 8.5%, range: − 17.7–89.1%).

Net capital stock in agriculture increased most in lower-middle-income countries (aggregated: 162.5%, mean: 120.2%, range: 11.8–204.3%), followed by upper-middle-income countries (aggregated: 146.2%, mean: 112.0%, range: 5.0–384.6%), and high-income countries (aggregated: 32.4%, mean: 56.5%, range: 4.2–151.2%).

**Figure 2:**
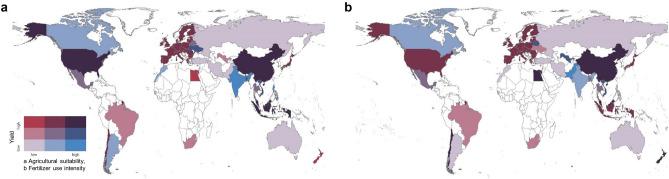
Bivariate choropleth maps of selected coproduction factors plotted against yield for the year 2014. Values were split into three tertiles; low, medium, high. (**a**) Agricultural suitability, (**b**) Fertilizer use intensity. Countries in white have no data for this year. Figure was created in R (version 3.6.1, https://www.r-project.org/) based on^58^.

Agricultural suitability and fertilizer use intensity for the year 2014 are plotted against yield in Fig. 2. These maps highlight how relative levels of each factor align with levels of yield per country or region. For instance, the European Union as a high yield region has medium levels of agricultural suitability and fertilizer use intensity (Fig. 2). On the other hand, China, as a high yield country, has high levels of agricultural suitability and fertilizer use intensity. India and the Philippines are the two countries that have relatively high agricultural suitability but relatively low yields. Pakistan, for instance, has high fertilizer use intensity, but relatively low yields.

### Model of yield as a function of anthropogenic and natural contributions

Standardized coefficients of the multiple regression model are reported in Table 1. The explanatory power of the model was R^2^ = 0.62 (adjusted R^2^, n = 64 observations). As expected, agricultural suitability is positively associated with the yields of the 15 crops (*p* < 0.01). Our results also confirm that fertilizer use intensity is positively associated with yield (*p* < 0.001). Among all logged and standardised variables, this variable had the strongest positive effect on yield. The association of manure use intensity also had a positive association with yield (*p* < 0.01). Together, coefficients of these variables show the importance of anthropogenic contributions to the coproduction of crop yield. We found no significant main effects of net capital stock intensity on yield.

**Table 1:** Model results: coefficients of the independent variables.

H	Independent variables	Adjusted R^2^: 0.62 (n = 64 observations)
		Standardized regression coefficients^a^ (Standard error)
	Intercept	− 0.10 (0.08)
+	Agricultural suitability	0.46 (0.17) **
+	Fertilizer use intensity	0.65 (0.14) ***
+	Net capital stock intensity	0.06 (0.12)
+	Manure use intensity	0.34 (0.10) **
−	Agricultural suitability:Fertilizer use intensity	− 0.35 (0.29)
−	Agricultural suitability:Manure use intensity	− 0.11 (0.21)
+ / −	Agricultural suitability:Net capital stock intensity	− 0.06 (0.26)

Fertilizer use intensity and agricultural suitability are positively associated with yield.

^a^Significance levels: *p* < 0.1; *p < 0.05; **p < 0.01, ***p < 0.001.

We found no interaction effects (at significance level *p* < 0.05) of agricultural suitability and fertilizer use intensity, contrary to our expectation that an increase in one of these variables could decrease the effect of the other on yield. However, the interaction effect between agricultural suitability and fertilizer use intensity, albeit above common significance levels, was present and negative, which could point to the fact that an increase in one coproduction factor leads to a decrease of the other in its effect on yield.

## Discussion

We found evidence for increasing efficiency in the use of fertilizer and manure as coproduction factors. While yield increased over the time period (2000–2014), the use of nitrogen fertilizer and manure stagnated. This was most pronounced for high-income countries, many of which are known to have increased their fertilizer use efficiency^25,26^ and their ratio of outputs to inputs in general (total factor productivity^27,28^). Continued energy input to agriculture (largely driven by nitrogen fertilizer, machinery and fuels) has been described for Asia, Latin America, while energy input decreased in Europe and stagnated North America^1^. This is in line with the development we observe for net capital stock in agriculture, with stagnation in high-income countries and a continued increase in lower- and upper-middle countries.

Our findings for coproduction of 15 crops on a national level confirm the positive association of nitrogen fertilizer with yield, which is in line with earlier findings, for instance within the European Union^26^ and globally^29^. We found a positive association of agricultural suitability with yield across all countries. This confirms the importance of natural contributions (here: soil fertility, topography and climate) in the coproduction of crops. Soil fertility and quality, as a considerable part of suitability, has been found to have a positive correlation with yield in the European Union^26^. German et al.^30^ have also found strong correlations between different aspects of soil quality and yield across different agricultural systems in their systematic review. However, another systematic review on pairwise associations of ecosystem services by Lee and Lautenbach^31^ found no clear direction of association (i.e. both positive and negative associations) between soil formation and composition and food production, and even a negative association between soil formation and composition and biomass production (including fodder). Note, however, that the composite indicator for suitability which we used here also contains climatic factors (temperature, precipitation), which have been found to have differing effects on yields, depending on crop type and world region^29,32^. In addition, it contains irrigated areas.

The literature on coproduction has, so far, largely remained conceptual and is hardly operationalised (e.g.^7,8^), which might be due to the lack of data allowing to distinguish these processes. Data availability on agricultural resources is relatively high and hence an ideal showcase for an understanding coproduction. Here we have operationalized the analysis of coproduction of crops, and included anthropogenic contributions at different stages in the provision of ecosystem services^4^, namely the management of agricultural landscapes (through the independent variables net capital stock, manure and fertilizer use intensity) and the mobilization or harvest of crops (net capital stock intensity). Our study of coproduction of agricultural crops is related to studying (the effects of) input intensity (i.e., amount of different forms of anthropogenic capital per area^33^). Our analysis, in addition, includes natural contributions as inputs and also studies the interactions between different contributions. We found that modelling the final ecosystem service (crop production) as the dependent variable of different types of contributions as independent variables is a well-suited way to study coproduction that could be applied to other services.

While we did not find evidence that the effect of natural contributions (soil fertility, topography and climate) on yield decreases when more fertilizer is applied at common significance levels, we found a negative interaction effect between fertilizer use intensity and agricultural suitability. Future studies could test whether this interaction of natural and anthropogenic contributions in producing crops occurs at smaller scales. Moreover, research could look in more detail into studying the effect of agricultural intensification also need to consider aspects of human wellbeing (such as food security) as an outcome^34^. While we did not find evidence for substitutability of agricultural suitability we found positive effects of agricultural suitability as an independent variable. In line with ecological or sustainable intensification measures, soil fertility to enhance yield could, for instance, be promoted through conservation tillage, crop rotation and planting of cover crops^35^.

Despite the use of aggregated data, we were able to identify the effect of natural and anthropogenic contributions on the coproduction of crops at the national level with global coverage. We argue that aggregation here is a “requisite simplicity”^36^ that allows studying the complex problem of coproduction of crops across systems. In our analysis, we used aggregated country data, which neglects potential within-country spatial variation. This was necessary as some data is only collected at the national level in an aggregated form (production, fertilizer use intensity), while other data would allow for a more fine-grained, spatially explicit analysis (agricultural suitability). With more data available in the future, one could study spatial–temporal correlations between observations of a country over time. The lack of spatial information on many land-use intensity factors in particular at the global level is a well-known challenge^37^. The same lack of data holds for other potentially relevant natural contributions (e.g. animal pollination, biological pest control). While the models explained a considerable proportion of the variation of yield, we lack information on other relevant anthropogenic contributions like the application of pesticides, or fuels for machinery, or agricultural knowledge^3^. Furthermore, we merged all crops, which limits the ability to detect potential variations between crops. The dataset for agricultural suitability we used, however, accounts for long-term crop-specific growing-conditions^14^. We also used anthropogenic contributions that characterize agriculture in general at the national level, which might actually differ for the 15 crops as compared to all crops. Agricultural suitability contains irrigated areas and hence also anthropogenic contributions.

Our study paves the way for addressing further research questions. For instance, the effect of other important contributions to agricultural production, like pollination^38^ or biological pest control^39^ could be studied at large scales. These interactions would be of particular interest as agriculture both harms biodiversity contributing to these services and benefits from it (biodiversity-production mutualism^40^). Moreover, different levels of anthropogenic and natural coproduction factors that potentially replace each other in producing comparable amounts of crops also need to be studied in terms of associated benefits and costs including the environmental effects of applying fertilizer^41^ or the negative effect of intensification on biodiversity^42^. This also includes the socioeconomic effects of enhancing different levels of natural and anthropogenic contributions across farming systems on food security and profitability^43^.

## Conclusion

Yields of food, feed and energy crops depend on both natural and anthropogenic contributions. We have analysed coproduction of the yields of 15 major food, feed and energy crops globally on a national level. We found that crop-specific fertilizer use, manure use intensity and agricultural suitability for these crops explained variation in yields to a considerable degree. Our results contribute to a better understanding of how natural and anthropogenic contributions to agricultural yields interact. We found no significant interaction effect between agricultural suitability (including soil fertility, topography and climate) and fertilizer use intensity. The significant association of agricultural suitability with yield over all countries implies the importance of agricultural suitability, of which soil fertility could be enhanced through measures of ecological or sustainable intensification.

## Methods

We collected data for indicators for coproduction and yield of 15 crops for 67 countries and the European Union (including the United Kingdom) across the globe for four time steps (2000, 2006, 2010, and 2014). We excluded Croatia because of the lack of data for net capital stock intensity, and Belarus for the year 2000 because of inconsistently low reported values of fertilizer use. Choice of crops, countries and time steps was restricted by data availability of different indicators (see below). Data for all variables and time steps was available for 20 countries and the European Union, i.e. 47 countries in total (see Supplementary Information, Table S1).

Agricultural suitability: We used an aggregated suitability index, available for 15 major crops: barley, cassava, groundnut, maize, millet, oil palm, potato, rapeseed/canola, rice, rye, sorghum, soybean, sugarcane, sunflower, wheat (including summer and winter wheat)^14^. The index comprises crop-specific conditions for soil structure and fertility, climate and topography based on a fuzzy logic approach (for further details see^14^). It serves as an indicator for natural contributions to crop production, including the ecosystem services that contribute to soil fertility. Note that it also contains irrigated areas^14^, and hence indirectly includes anthropogenic contributions. The index contains average values for the period 1981–2010. In order to account for the spatial distribution of the 15 crops we used data on harvested area by Monfreda et al.^44^ to create a harvested area-weighed agricultural suitability index. The agricultural suitability dataset was resampled to the grid cell size of the Monfreda et al.^44^ dataset (5 min by 5 min) using the bilinear resampling method in ArcMap 10.7. For each grid cell we created a weight by dividing the total area harvested of the 15 crops by the respective sum across the country and then multiplied this weight with the agricultural suitability data. The resulting grid cell values were then summed by country.

**Figure 3:**
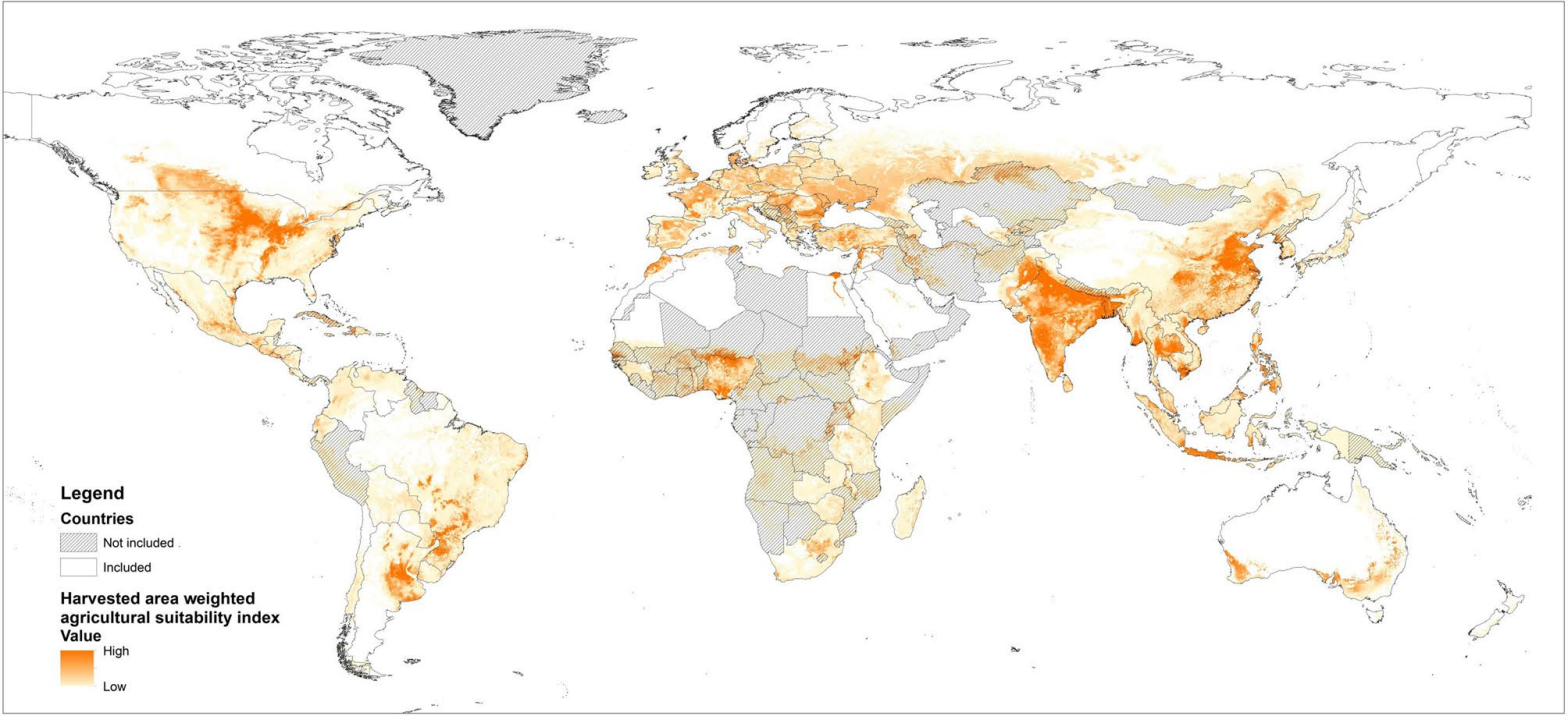
Global map of the harvested area weighted agricultural suitability index on cropland of the 15 selected crops (based on^14,44^). Countries included in the analysis are marked white, countries excluded (no reported crop-specific nitrogen use) are dashed. Figure was created in ArcMap (version 10.7, https://desktop.arcgis.com/en/arcmap/) based on^14,44,58^.

Fertilizer use intensity: We collected data on nitrogen fertilizer use by crops from the International Fertilizer Association IFA^45–48^. Crop-specific fertilizer application data was available for all 15 crops and for four time steps around 2000, 2006/07, 2010/11, 2014/15 (years slightly varying due to data collection and reporting method). This dataset determined the choice for the four time steps. For in total 67 countries and the European Union data was available for at least one time step (see Fig. 3 and Supplementary Information Table S1). According to the dataset these countries applied a large proportion of the global artificial nitrogen fertilizer, namely 92.4% (2006/07), 94.3% (2010/11) and 93.6% (2014/15). The selected countries accounted for 80.9% (2006), 81.3% (2010) and 81.7% (2014) of the caloric value of the production of these crops (own calculations based on yields reported by^49^).

Net capital stock intensity: We took annual data of net capital stock (i.e. fixed capital, accounting for depreciation, used in agriculture, including machinery, buildings and equipment) from FAOSTAT for the selected countries and years (2000, 2006, 2010, 2014^50^). This data was used as a composite measure for anthropogenic capital used as an input to produce crops. As this data is not crop-specific we made the assumption that the intensity of capital stock in agriculture per area harvested as an average for all crops also holds for the 15 selected crops. That is, we divided net capital stock in agriculture by the total area harvested for all crops to get an average intensity measure. Values were transformed to 2014 US$ values using inflation factors^51^.

Manure use intensity: We took annual data on manure applied to soils from FAOSTAT for the selected countries and years (2000, 2006, 2010, 2014)^52^. As this data is not crop-specific we made the assumption that manure applied to soils is used proportionally to area harvested. That is, we multiplied the value for each country by the fraction of area harvested of the selected 15 crops of the total area harvested per year and then divided this value by area harvested for the 15 crops to get an intensity value.

Yield: Yield was calculated as production per area harvested. Data on annual crop production and the respective area harvested was taken from FAOSTAT for the selected countries, crops and years (2000, 2006, 2010, 2014)^49^. All crop production data was transformed to caloric values from tonnes to kJ based on FAO^53^. We then summed the values of the 15 crops for each country or region and year.

To visualise trajectories of three anthropogenic coproduction factors against yield we summed the values for production, fertilizer use, manure use and net capital stock in agriculture, each divided by the sum of total area harvested for countries belonging to the same income class according to the Worldbank (“lower-middle”, “upper-middle” and “high” income)^54^ and for which data was available for each of the four time steps. This was the case for seven of 24 lower-middle income countries, seven of 19 upper-middle income countries, and seven of 17 high income countries (including the EU as one region) considered in this analysis (see Supplementary Information Table S1). We reported changes relative to the values for the year 2000 (100%). In addition we calculated the mean values and the minimum and maximum values of all countries belonging to the same income class.

We used the statistical software package R 3.6.1^55^ run via RStudio 1.2.1335^56^ for data analysis. We used linear regression model predicts yield as a function of anthropogenic and natural coproduction factors. We also included interaction effects with the independent variable agricultural suitability to test for enhancement or substitution effects between natural and anthropogenic coproduction factors. Hypothesised main and interaction effects are found in Table 2. For the linear regression model all variables were available for each country for one, two, three or four years. We took the mean of the variables fertilizer use intensity, net capital stock intensity, manure use intensity and years over the available years per country to reduce the risk of pseudoreplication of observations of one country. The linear model was:

**Table 2:** Explanatory variables and hypotheses.

Variable	Hypothesised effect on yield
**Main effects**	
Agricultural suitability (index on climate, soil, topography, i.e. natural coproduction factors)	+ A higher agricultural suitability leads to higher yields
Fertilizer use intensity (anthropogenic coproduction factor)	+ Higher fertilizer use intensity leads to higher yields
Manure use intensity (anthropogenic coproduction factor)	+ A higher manure use intensity leads to higher yields
Net capital stock intensity (anthropogenic coproduction factor) in 2014 US$	+ A higher proportion of net capital stock/area leads to higher yields due to more industrialised agriculture
**Interaction effects**	
Agricultural suitability:fertilizer use intensity	− A substitution effect, i.e. the higher fertilizer use intensity the lower the effect of suitability on yield
Agricultural suitability:manure use intensity	− A substitution effect, i.e. the higher the amount of manure applied to soils the lower the effect of suitability on yield
Agricultural suitability:net capital stock intensity	+ / − An enhancement effect, i.e. the higher the amount of net capital stock, the more effective a country can make use of agricultural suitability for achieving higher yields (e.g., using machinery for less suitable soils); or a substitution effect

Yield = f(agricultural suitability + fertilizer use intensity + net capital stock intensity + manure use intensity + agricultural suitability:fertilizer use intensity + agricultural suitability:net capital stock intensity + agricultural suitability:manure use intensity).

We checked outliers with the help of the function *olsrr: ols_plot_cooksd_bar* in R and identified four countries that we then removed from the dataset due to high Cook’s distances^57^: Azerbaijan, Egypt, Kuwait, and Malaysia. We tested the model for collinearity. None of the explanatory variables were highly correlated (Pearson's r between fertilizer use intensity and net capital stock intensity was *p* = 0.705, and both were kept in the model).

To equalize spread and reduce leverage, we log-transformed all variables. We scaled variables (mean-centred and divided by their standard deviations) to make regression coefficients comparable. We used the number of observations per country as weights in the linear model. Code for data preparation and analysis as well as datasets used are available (see data availability statement).

## Supplementary Information

Supplementary Information.

## Data Availability

Data and code are available at https://github.com/matthiasschroeter/Coproduction_crops.
