# Supplementary material for: Distinguishing anthropogenic and natural contributions to coproduction of national crop yields globally
Source: Sci Rep. 2021 May 24;11:10821. doi: 10.1038/s41598-021-90340-1 (PMC8144206; doi:10.1038/s41598-021-90340-1)
Supplement: Supplementary file 1 — Supplementary Information. [file 41598_2021_90340_MOESM1_ESM.pdf]

Supplementary Information

Distinguishing anthropogenic and natural contributions to coproduction of national crop yields globally

Authors: Matthias Schröter, Lukas Egli, Lilith Brüning, Ralf Seppelt

Table S1: Countries included in the analysis with data availability for the respective years (2000, 2006, 2010, 2014) and Worldbank income group.

Country name	2000	2006	2010	2014	Income group
Albania	X				Upper-middle
Algeria	X				Upper-middle
Argentina	X	X	X	X	High
Australia	X	X	X	X	High
Azerbaijan	X				Upper-middle
Bangladesh	X	X	X	X	Lower-middle
Belarus			X	X	Upper-middle
Bolivia (Plurinational_State_of)	X				Lower-middle
Brazil	X	X	X	X	Upper-middle
Cambodia	X				Low
Canada	X	X	X	X	High
Chile	X	X	X	X	High
China,_mainland	X	X	X	X	Upper-middle
Colombia	X				Upper-middle
Costa_Rica	X				Upper-middle
Dominican_Republic	X				Upper-middle
Ecuador	X				Upper-middle
Egypt	X	X	X	X	Lower-middle
El_Salvador	X				Lower-middle
Ethiopia	X				Low
Guatemala	X				Lower-middle
Guinea	X				Low
Honduras	X				Lower-middle

India	X	X	X	X	Lower-middle
Indonesia	X	X	X	X	Lower-middle
Iran_(Islamic_Republic_of)		X	X	X	Upper-middle
Israel	X				High
Japan	X	X	X	X	High
Jordan	X				Upper-middle
Kenya	X				Lower-middle
Kuwait	X				High
Lao_People's_Democratic_Republic	X				Lower-middle
Lebanon	X				Upper-middle
Madagascar	X				Low
Malawi	X				Low
Malaysia	X	X	X	X	Upper-middle
Mauritania	X				Lower-middle
Mexico	X	X	X	X	Upper-middle
Morocco	X	X	X	X	Lower-middle
Myanmar	X				Lower-middle
New_Zealand	X			X	High
Nicaragua	X				Lower-middle
Nigeria	X				Lower-middle
Norway	X				High
Pakistan	X	X	X	X	Lower-middle
Paraguay	X				Upper-middle
Philippines	X	X	X	X	Lower-middle
Republic_of_Korea	X				High
Republic_of_Moldova	X				Lower-middle
Russian Federation		X	X	X	High
Saudi_Arabia	X				High
South_Africa	X	X	X	X	Upper-middle
Sri_Lanka	X				Lower-middle
Switzerland	X				High

Syrian_Arab_Republic	X				Lower-middle
Thailand	X	X	X	X	Upper-middle
Togo	X				Low
Turkey	X	X	X	X	Upper-middle
Ukraine		X	X	X	Lower-middle
United_Republic_of_Tanzania	X				Low
United_States_of_America	X	X	X	X	High
Uruguay	X				High
Uzbekistan		X	X	X	Lower-middle
Venezuela_(Bolivarian_Republic _of)	X				High
Viet_Nam	X	X	X	X	Lower-middle
Zambia	X				Lower-middle
Zimbabwe	X				Low
EU27	X	X	X	X	High
